# Improvement of Background Solution for Optically Induced Dielectrophoresis-Based Cell Manipulation in a Microfluidic System

**DOI:** 10.3389/fbioe.2021.759205

**Published:** 2021-11-22

**Authors:** Po-Yu Chu, Chia-Hsun Hsieh, Chih-Yu Chen, Min-Hsien Wu

**Affiliations:** ^1^ Ph.D. Program in Biomedical Engineering, Chang Gung University, Taoyuan City, Taiwan; ^2^ Division of Hematology-Oncology, Department of Internal Medicine, New Taipei Municipal TuCheng Hospital, New Taipei City, Taiwan; ^3^ Division of Hematology-Oncology, Department of Internal Medicine, Chang Gung Memorial Hospital at Linkou, Taoyuan City, Taiwan; ^4^ Collage of Medicine, Chang Gung University, Taoyuan City, Taiwan; ^5^ Graduate Institute of Biomedical Engineering, Chang Gung University, Taoyuan City, Taiwan; ^6^ Department of Chemical Engineering, Ming Chi University of Technology, New Taipei City, Taiwan

**Keywords:** optically induced dielectrophoresis, cell manipulation, microfluidic technology, sucrose solution, low conductivity

## Abstract

Optically induced dielectrophoresis (ODEP) is effective for cell manipulation. However, its utilization has been limited by the requirement of solution with low conductivity. This issue has been ignored in ODEP-relevant studies. To address this issue, this study aims to investigate to what extent the cell viability and performance of ODEP-based cell manipulation are affected by low conductivity conditions. Additionally, this study aims to modify sucrose solutions to reduce the impacts caused by low-conductivity solutions. Results revealed the use of sucrose solution in ODEP operation could significantly reduce the viability of the manipulated cells by 9.1 and 38.5% after 2- and 4-h incubation, respectively. Prolonged operation time (e.g., 4 h) in sucrose solution could lead to significantly inferior performance of cell manipulation, including 47.2% reduction of ODEP manipulation velocity and 44.4% loss of the cells manipulatable by ODEP. The key finding of this study is that the use of bovine serum albumin (BSA)-supplemented sucrose solution (conductivity: 25–50 μS cm^−1^) might significantly increase the cell viability by 10.9–14.8% compared with that in sucrose solution after 4 h incubation. Moreover, the ODEP manipulation velocity of cells in the BSA-supplemented sucrose solution (conductivity: 25 μS cm^−1^) was comparable to that in sucrose solution during 4-h incubation. More importantly, compared with sucrose solution, the use of BSA-supplemented sucrose solution (conductivity: 25–50 μS cm^−1^) contributed high percentage (80.4–93.5%) of the cells manipulatable by ODEP during 4-h incubation. Overall, this study has provided some fundamental information relevant to the improvement of background solutions for ODEP-based cell manipulation.

## Introduction

In biological research, the manipulation of cells has a wide variety of applications [e.g., cell isolation and purification (Gupta et al., 2012; Li et al., 2015; Song et al., 2015; Chiu et al., 2016; L; D’Amico et al., 2017; Liao et al., 2018; Chu et al., 2019a; Sasan; Asiaei et al., 2019; Chu et al., 2020b; Wang et al., 2020), cell alignment and patterning (Puttaswamy et al., 2010; Lin et al., 2012), biosensing (Fatima H Labeed et al., 2003; Hoettges et al., 2007; Bambardekar et al., 2015; Song et al., 2015; Chu et al., 2019a; Wang et al., 2020), diagnostic use (Chiu et al., 2016; Liao et al., 2018; Chu et al., 2019a; Wang et al., 2020), or tissue engineering (Puttaswamy et al., 2010; Lin et al., 2012; Bambardekar et al., 2015; Song et al., 2015)]. However, the precise manipulation of biological cells has been a technical challenge due mainly to the lack of a corresponding approach capable of fine manipulation of such microscale substances. Thanks to the recent progress in Bio-MEMS (Bio-Micro-Electromechanical System) and microfluidic technology, several innovative techniques such as thermophoresis (Asiaei et al., 2019), acoustophoresis (Li et al., 2015), optical tweezers (Bambardekar et al., 2015), dielectrophoretic (DEP) force (Fatima H Labeed et al., 2003; Hoettges et al., 2007; Srinivasu Valagerahally Puttaswamy et al., 2010; Gupta et al., 2012; Song et al., 2015; L D'Amico et al., 2017), and optically induced dielectrophoresis (ODEP) (Lin et al., 2012; Chiu et al., 2016; Liao et al., 2018; Chu et al., 2019a; Chu et al., 2020b; Wang et al., 2020), have been incorporated in a microfluidic system for various cell manipulation applications. Among these techniques, the heat damage caused by thermo-, or acoustophoresis-, or optical tweezer-based approaches during cell manipulation might be lethal to biological cells (Neuman et al., 1999; Li et al., 2015; Lin et al., 2018). The integration of a DEP force-based mechanism into a microfluidic system is believed to be an effective method for cell manipulation (Gupta et al., 2012; Song et al., 2015). Reports in the literature have also demonstrated the use of this technique for a wide variety of biological applications (e.g., rapid trapping and sorting of cells or microorganisms (Gupta et al., 2012; L; D'Amico et al., 2017), identification of antitumor drugs or antibiotic resistance (Fatima H Labeed et al., 2003; Hoettges et al., 2007), or classification of cell differentiation (Song et al., 2015)). However, DEP force-based cell manipulation normally requires precise microfabrication to create a metal electrode layout that is specific to applications. This requirement is, to some extent, costly, time-consuming, and technically-demanding.

To tackle the technical hurdle, the incorporation of the ODEP mechanism into a microfluidic system facilitates the ability of scientists to quickly alter the “virtual” electrode layout for a specific cell manipulation purpose simply via the manipulation of optical images projecting on the cells. This technical feature not only largely reduces the requirement of microfabrication, but also makes the operation of cell manipulation more flexible, and user-friendly through computer-interfaced control (Chiou et al., 2005; Chu et al., 2020b). Leveraging the technical merits of ODEP-based cell manipulation, this technical feature has been successfully demonstrated to manipulate biological cells for various applications [e.g., cell alignment and patterning for tissue engineering (Lin et al., 2012), collection of high-purity circulating tumor cells (CTCs) of different types (Chiu et al., 2016; Liao et al., 2018; Chu et al., 2020b), or evaluation of antitumor drug or antibiotic resistance for cancer cells or microorganisms (Chu et al., 2019a; Wang et al., 2020)]. The utilization of the ODEP mechanism for microparticle manipulation was first proposed in 2005 (Chiou et al., 2005). Briefly, an alternating-current (AC) voltage is first exerted in the top and bottom substrates [e.g., indium-tin-oxide (ITO) glass substrates] of an ODEP system to generate a uniform electric field in the thin solution layer sandwiched between the two substrates. In this situation, the microparticles suspended in the solution are electrically polarized. When the bottom photoconductive substrate (e.g., ITO glass with a coating layer of photoconductive material) of the ODEP system is illuminated, the projected light could excite electron-hole pairs and thus significantly decrease the electrical impedance of the light-illuminated area. This phenomenon will lead to the exerted electric voltage dropping across the solution layer inside the light-illuminated area. This phenomenon, in turn, creates a locally nonuniform electric field in the light-illuminated area. In ODEP practice, the interaction between the nonuniform electric field and electrically polarized microparticles (e.g., biological cells) is practically used to manipulate these microparticles. In operation, therefore, scientists can simply control the light images projected into an ODEP system to manipulate the biological cells in a controllable manner (Chiou et al., 2005; Chu et al., 2020b).

Although an effective or precise manipulation of cells can be achieved using the ODEP-based technique, there is one critical technical issue that should be further addressed. ODEP-based microparticle manipulation normally requires the background solution, in which the microparticles are suspended, with low conductivity. This is mainly due to the environment of a high conductivity solution could attenuate the ODEP force acting on a microparticle, which in turn largely affects the ODEP-based microparticle manipulation (Valley et al., 2008). In practice, for example, ODEP-based cell manipulation was commonly carried out in the sucrose solution [9.5% (w/v)] with low conductivity (e.g., 6.9 μS cm^−1^) (Liao et al., 2018; Chu et al., 2019a; Chu et al., 2020a; Chu et al., 2020b). However, this solution condition might not be friendly to general biological cells. The biochemical activities occurring within a living cell are well recognized to normally require the participation of various chemicals, biomolecules, or ions (Yao and Asayama, 2017), which could accordingly lead to a high conductivity environment (e.g., conductivity of commercially-available RPMI cell culture medium: 11,770 μS cm^−1^). As a result, the background solution with low conductivity required for ODEP-based cell manipulation could affect the properties (e.g., cell viability) of the manipulated cells. This phenomenon could in turn affect the performance of ODEP-based cell manipulation as the cell membrane integrity of biological cells could affect their ODEP manipulation force, as reported in our previous study (Chu et al., 2019a; Chu et al., 2019b). Overall, the requirement of a low conductivity environment in ODEP-based cell manipulation could greatly hinder the widespread application of the ODEP technique for biological research. Nevertheless, this issue has been generally ignored in ODEP-relevant studies.

To address this issue, this study aims to investigate to what extent the cell viability of cells as well as the performance (e.g., the ODEP manipulation velocity or the percentage of the cells manipulatable by ODEP force) of ODEP-based cell manipulation are affected by the use of a background solution with low conductivity (e.g., 6.9 μS cm^−1^) in ODEP operation. In addition, this study also aims to explore the possibility of modifying the sucrose solution by supplementation with other ingredients [e.g., dextrose, cell culture medium, bovine serum albumin (BSA)] to reduce the impact of the low conductivity condition of the background solution on the viability of the manipulated cells and the performance of ODEP-based cell manipulation. The results showed that ODEP-based cell manipulation using 9.5% sucrose solution could significantly compromise the viability of the manipulated cells by 9.1 and 38.5% after 2 and 4 h of incubation, respectively. Prolonged operation time (e.g., 4 h) in sucrose solution could significantly lead to inferior performance of ODEP-based cell manipulation, including 47.2% reduction of ODEP manipulation velocity and 44.4% loss of the cells manipulatable by ODEP force compared with them in the initial stage of ODEP-based cell manipulation. The key finding of this study is that the use of BSA-supplemented sucrose solution with a conductivity range of 25–50 μS cm^−1^ might significantly increase the cell viability by 10.9–14.8% compared with the cell viability in sucrose solution after 4 h of incubation. Moreover, the ODEP manipulation velocity of cells in the BSA-supplemented sucrose solution (conductivity: 25 μS cm^−1^) was comparable to the ODEP manipulation velocity of cells in the conventional sucrose solution during a 4-h incubation. Compared with the use of conventional sucrose solution, more importantly, the use of BSA-supplemented sucrose solution (conductivity: 25–50 μS cm^−1^) could contribute to the high percentage (80.4–93.5%) of the cells manipulatable by ODEP force during 4 h of incubation, which also showed no significant difference compared with that in the initial stage of ODEP-based cell manipulation in the sucrose solution. As a whole, this study has provided some fundamental information relevant to the improvement of background solutions for ODEP-based cell manipulation.

## Materials and Methods

### ODEP Microfluidic Chip and Experimental Setup

**FIGURE 1:**
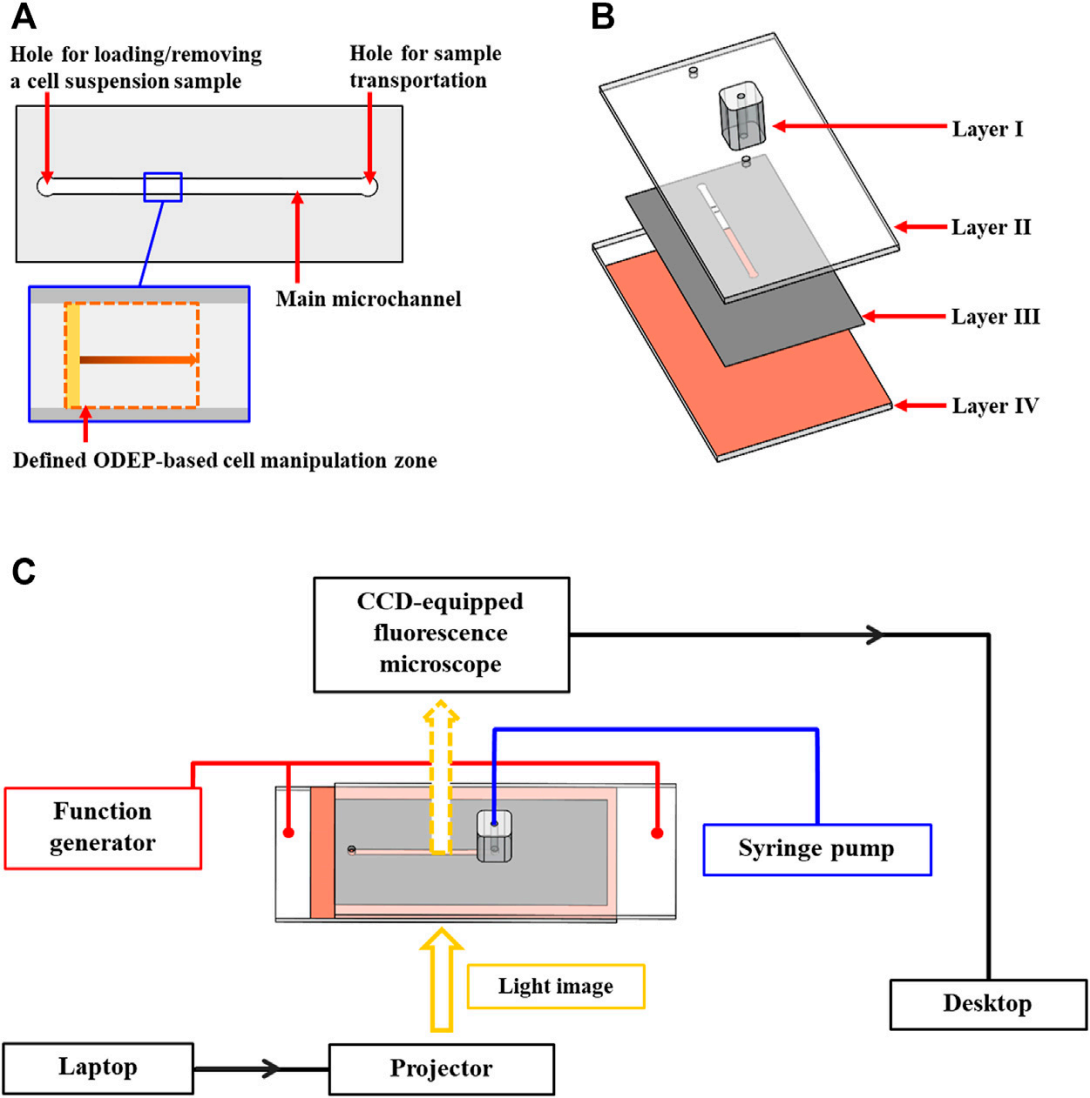
Schematic presentation of the **(A)** top-view layout and **(B)** structure of the ODEP microfluidic chip (Layer I, PDMS connector; Layer II, ITO glass; Layer III, double-sided adhesive tape with a fabricated microchannel; Layer IV, ITO glass coating with a photoconductive material, and **(C)** overall experimental setup.

In this study, an ODEP microfluidic chip with its top-view layout illustrated in Figure 1A was used to evaluate the performance of ODEP-based cell manipulation. In the microfluidic chip, briefly, a main microchannel (L: 20.0 mm, W: 1.0 mm, H: 50.0 µm) with two holes (D: 1.0 mm) was designed for loading/removing a cell suspension sample as well as the sample transportation. In this design, ODEP-based cell manipulation was carried out in the defined ODEP-based cell manipulation zone (L: 0.8 mm, W: 1.0 mm) (Figure 1A) in the main microchannel. The structure of the ODEP microfluidic chip is schematically illustrated in Figure 1B, encompasses a fabricated polydimethylsiloxane (PDMS) connector (Layer I), an indium-tin-oxide (ITO) glass (Layer II), a double-sided adhesive tape (thickness: 50 µm) with a fabricated microchannel (Layer III), and an ITO glass coating with a photoconductive material containing a 10-nm-thick molybdenum layer and a 1-μm-thick hydrogenated amorphous silicon layer (Layer IV). The fabrication of the ODEP microfluidic chip was based on computer-numerical-controlled (CNC) machining for mold making (for Layer I), a metal mold-punching fabrication process (for Layer III), PDMS replica molding (for Layer I), and a thin-film technology using sputtering and plasma-enhanced chemical vapor deposition (PECVD) (for Layer IV) as described previously (Chu et al., 2020a; Chu et al., 2020b). After each layer was fabricated, the two through-holes located in Layer II were connected directly with a microchannel in Layer III (Figure 1B), followed by assembly with Layer IV with the aid of double-sided adhesive tape (Layer III). In this work, the PDMS connector (Layer I) was bonded onto one of the through-holes in Layer II via the surface treatment of plasma oxidation. In this work, the tested cell suspension sample was transported in the main microchannel using a syringe pump (Figure 1C). For ODEP-based cell manipulation, a function generator (AFG-2125, Good Will Instrument Co., Ltd., New Taipei City, TW) was used to apply an alternating current (AC) between the two ITO glasses (i.e., Layers II and IV; Figure 1B) of the ODEP microfluidic chip. A commercial digital projector (EB-X05, Epson, Nagano, JP) coupled with a computer was used to illuminate light images onto the photoconductive material (Layer IV; Figure 1B) to generate ODEP force on the manipulated cells. In this study, a CCD-equipped fluorescence microscope (Zoom 160, OPTEM, US) was utilized to observe and record the performance of ODEP-based cell manipulation. The overall experimental setup is schematically illustrated in Figure 1C (the photograph of the overall experimental setup was provided in Supplementary Figure S1).

### Performance Evaluation of ODEP-Based Cell Manipulation

The working principle of ODEP for cell manipulation has been described in the introduction section. In theory, the ODEP force generated on a biological cell can be expressed by Eq. 1 (Valley et al., 2008; Chu et al., 2019a; Chu et al., 2020a):

$$F_{\mathrm{DEP}}\;=\;2\pi r^{3}\varepsilon_{0}\varepsilon_{m}\text{Re}\left[f_{\mathrm{CM}}\right]\nabla \text{|}E{\text{|}}^{2}$$ (1)

Based on Eq. 1, *r* (cellular radius), *ε*_*0*_ (vacuum permittivity), *ε*_*m*_ (relative permittivity of the surrounding solution), ∇ |*E*|^2^ (gradient of electric field squared), and Re [*f*_*CM*_] [real part of the Clausius–Mossotti factor (*f*_*CM*_)] are the factors that determine the ODEP force acting on a cell (Valley et al., 2008; Chu et al., 2019a; Chu et al., 2020a). In Eq. 1, the *f*_*CM*_ can be further described by Eq. 2 (Valley et al., 2008; Chu et al., 2020a):

$$f_{\mathrm{CM}}=\frac{\varepsilon_{\mathrm{cell}}*-\varepsilon_{m}*}{\varepsilon_{\mathrm{cell}}*+2\varepsilon_{m}*}\;$$ (2)

In Eq. 2, $\varepsilon_{cell}^{*}$ and $\varepsilon_{m}^{*}$ represent the complex permittivity of the cell, and surrounding solution, respectively. Taken together [Eqs 1, 2, the electric properties of the background solution [i.e., $\varepsilon_{m}^{*}$ of Eq. 2] and the manipulated cell [i.e., $\varepsilon_{cell}^{*}$ of Eq. 2] play important roles in the ODEP force generated on a particular cell under a given size of cell and given electric conditions (i.e., the magnitude and frequency of the electric voltage applied) (Valley et al., 2008; Chu et al., 2020a).

In this study, therefore, the impact of solution conductivity on the performances of ODEP-based cell manipulation including the ODEP force generated on a cell, as well as the percentage of the cells manipulatable by the ODEP force, were evaluated experimentally. For the former, the ODEP manipulation force (a net force between the ODEP force and friction force, acting on the manipulated cell) was then experimentally evaluated based on the method described previously (Chu et al., 2019a; Chu et al., 2020a; Chu et al., 2020b). Based on a steady state condition, briefly, the ODEP manipulation force acting on a cell is balanced by the viscous drag of fluid exerted on such a cell under a continuous flow condition. The hydrodynamic drag force of a moving cell was therefore used to assess the ODEP manipulation force of a cell according to Stokes’ law (Eq. 3):

F = 6πrηv (3)

**FIGURE 2:**
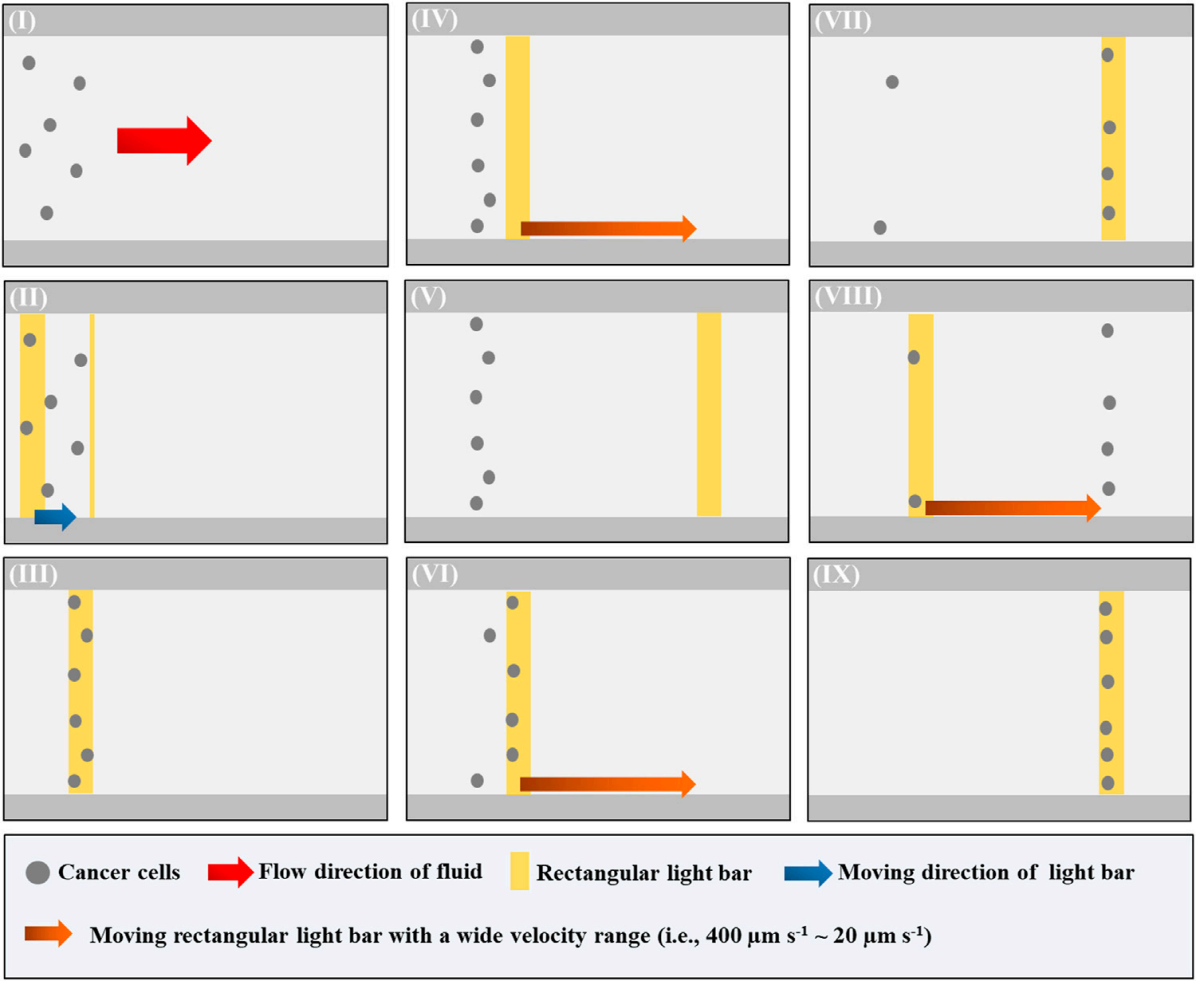
Schematic presentation of the operating procedures for the evaluation of “maximum velocity” of the dynamic light image that can manipulate cells (or ODEP manipulation velocity): (I) a cell suspension sample was loaded and then transported to the upstream area of the ODEP-based cell manipulation zone via fluid control, (II–III) the cells tested were moved to the start line of ODEP-based cell manipulation zone using a moving rectangular light bar, (IV–V) a moving rectangular light bar with a high moving velocity (e.g., 400 μm s^−1^) was first used to manipulate the cells. If no cell was effectively manipulated, the moving velocity of the light bar used for the next round of screening was progressively reduced (set decrement: 20 μm s^−1^), (VI–VII) once a cell was able to be manipulated by the moving light image with a particular moving velocity. This velocity was regarded as the “maximum velocity” of the dynamic light image that can manipulate cells (i.e., ODEP manipulation velocity), and (VIII–IX) the “screening process” [i.e., (IV–VII)] was continuously performed at a set velocity decrement of 20 μm s^−1^ until all the tested cells were effectively manipulated (a video clip is provided as a Supplementary Material).

In Eq. 3, *r* (cellular radius)*, η* (fluidic viscosity)*,* and *v* (the velocity of a moving cell) are the parameters determining the ODEP manipulation force. Under the given solution and cellular size conditions, overall, the ODEP manipulation force of the manipulated cell can then be experimentally evaluated via the measurement of the maximum velocity of a moving light image that can manipulate such a cell (or called ODEP manipulation velocity in this study) (Chu et al., 2019a; Chu et al., 2020a; Chu et al., 2020b). The overall operating procedures for the measurement of ODEP manipulation velocity are illustrated in Figure 2. In operation, the cell suspension sample tested was loaded into the main microchannel and then transported to the upstream area of the ODEP-based cell manipulation zone using a syringe pump (Figure 1C), as shown in Figure 2I, soon followed by moving the cells tested to the start line of the ODEP-based cell manipulation zone using a moving rectangular light bar (L: 1.0 mm, W: 100.0 μm, V: 10 μm s^−1^) under the set magnitude (10 Vpp) and frequency (2 MHz) of the electric field for ODEP operation (Figures 2II–III). Then, a moving rectangular light bar with a wide velocity range (i.e., 400 μm s^−1^ ~ 20 μm s^−1^) was designed to “screen” the cells tested to determine the “maximum velocities” of a light bar image that can effectively manipulate the tested cells. In operation, a rectangular light bar with a high moving velocity (e.g., 400 μm s^−1^) was first used to manipulate the cells (Figures 2IV–V). If no cell was effectively manipulated (Figure 2V ), the moving velocity of the light bar used for the next round of screening was progressively reduced (set decrement: 20 μm s^−1^). Once a cell was able to be manipulated by the moving light image with a particular moving velocity (Figures 2VI–VII), this velocity was regarded as the “maximum velocity” of the dynamic light image that can manipulate cells (i.e., ODEP manipulation velocity). Then, the abovementioned “screening process” (Figures 2IV–VII) was continuously performed at a set velocity decrement of 20 μm s^−1^ until all the tested cells were effectively manipulated (Figures 2VIII–IX). In every round of the “screening” process, the number of cells which were unable to be manipulated by moving light images was then recorded. Based on this measurement, the percentages of the cells manipulatable by ODEP force [i.e., (total cell number-the number of the cells unable to be manipulated)/(total cell number) * 100%] under a particular operating condition were then obtained.

### Evaluation of Cell Viability of the Cells Treated With Different Background Solutions

To determine to what extent the viability of biological cells is affected by background solutions with different compositions or conductivity, the following experimental work was carried out. In this work, the PC-3 cancer cell line, one of the commonly-used cancer cell lines in cancer-related studies (Chiu et al., 2016; Liao et al., 2018; Chu et al., 2020a), was used as a model cell for the tests. Briefly, 10^5^ PC-3 cancer cells were treated with 50 μl different background solutions at 37°C for 0, 2, and 4 h within this time frame, and ODEP-based cell manipulation was commonly carried out based on our previous experience (Chiu et al., 2016; Chu et al., 2020a). Then, the background solution of the treated PC-3 cell suspension was replaced by phosphate-buffered saline (PBS) (UNI-ONWARD corp., New Taipei City, TW). The treated PC-3 cancer cells were then stained with 1.0 μg ml^−1^ PI (propidium iodide) fluorescent dye (Invitrogen, CA, US) for 15 min for the identification of live (nonstained) and cell membrane-damaged (red staining) PC-3 cancer cells, followed by the quantification of live and dead PC-3 cancer cells using a flow cytometer (CytoFLEX, Beckman Coulter, CA, US) (Liu et al., 2017). Based on cell enumeration, the percentage of viable cells [i.e., (the number of live cells)/(total cell number) *100%] was then evaluated.

### Preparation of Background Solutions

In this study, 9.5% (w/v) sucrose solution (Merck, Darmstadt, DE) was used as the basic solution, as it is commonly used in several ODEP-based cell manipulations (Liao et al., 2018; Chu et al., 2019a; Chu et al., 2020a; Chu et al., 2020b). Thus, the background solutions with different properties (e.g., conductivity) were prepared by adding the basic solution to other ingredients [e.g., dextrose (Thermo Fisher Scientific, Waltham, MA, US), RPMI cell culture medium (Thermo Fisher Scientific, Waltham, MA, US), and BSA (bovine serum albumin; Sigma-Aldrich, MO, US)]. In addition, 5% (w/v) mannitol solution (OPS Diagnostics, Lebanon, NJ, US), a commonly used DEP background solution, was also used for ODEP-based cell manipulation tests in this study (Archer et al., 1999; Hoettges et al., 2007; L; D'Amico et al., 2017). The detailed recipes are summarized in Table 1. After the preparation of background solutions, the conductivity, osmolarity, and pH of these solutions were then measured using a Consort c5010 (Consort BVBA, Turnhout, BE), OSMOMAT 3000 (Gonotec, DE), and pH meter (SP-701, Suntex, Taipei, TW), respectively. All the measurements were carried out at 25°C.

**TABLE 1:** The composition and properties of the background solutions prepared for this study.

**Solution type**	**Composition**	**Conducitity (μS cm** ^**−1**^)	**Osmolarity (mOsmol kg** ^**−1**^)	**pH**
Sucrose	9.5% w/v Sucrose	6.9	285	6.48
M	5% w/v Mannitol	1.6	280	6.93
SD	8.5% w/v Sucrose and 0.3% w/v Dextrose	5.8	277	6.32
SR25	9.5% w/v Sucrose and 0.1% v/v RPMI	24.5	291	6.20
SR50	9.5% w/v Sucrose and 0.3% v/v RPMI	50.4	290	6.24
SB25	9.5% w/v Sucrose and 0.25% w/v BSA	25.5	291	6.52
SB50	9.5% w/v Sucrose and 0.5% w/v BSA	50.2	292	6.65

### Statistical Analysis

Data are presented as the mean ± the standard deviation. One-way ANOVA was used to assess the impact of the tested experimental conditions on the results. Tukey’s honestly significant difference (HSD) post hoc test was used to compare differences between the two conditions tested when the null hypothesis of the ANOVA was rejected.

## Results and Discussion

### Effect of the Sucrose Solution (9.5%) on the Cell Viability and the Performance of ODEP-Based Cell Manipulation

**FIGURE 3:**
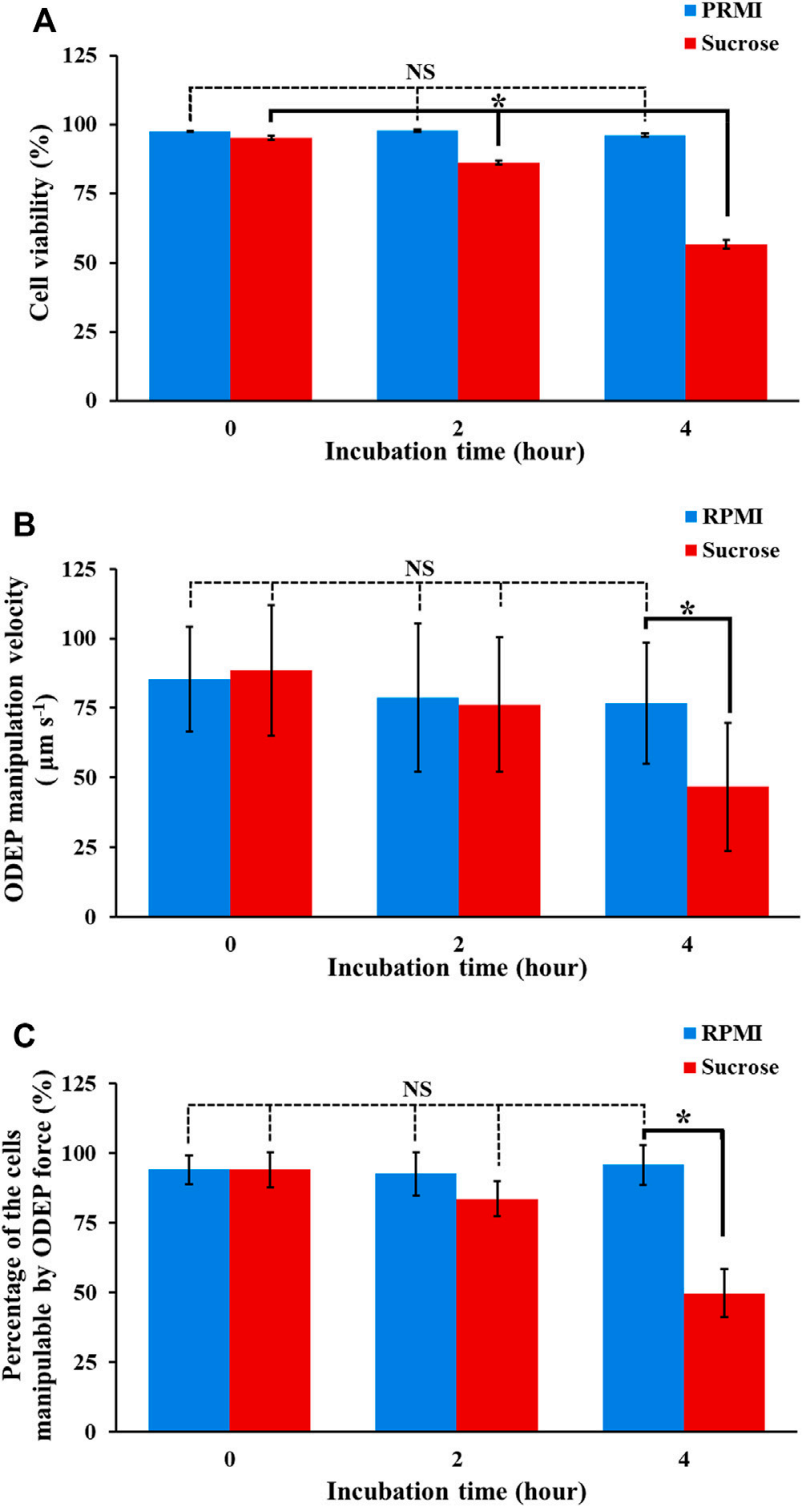
The **(A)** cell viability, **(B)** ODEP manipulation velocity, and **(C)** percentage of the cells manipulatable by ODEP force of the PC-3 cancer cells treated with RPMI culture medium and sucrose solution (9.5%) for 0, 2, and 4 h. The results are presented as the mean ± standard deviation of at least 3 separate experiments. [NS, no significance and *, significant difference (*p* < 0.05)].

Reports in the literature have demonstrated the utilization of ODEP-based cell manipulation for various applications (Lin et al., 2012; Chiu et al., 2016; Liao et al., 2018; Chu et al., 2019a; Chu et al., 2020b; Wang et al., 2020). In these studies, a sucrose solution with a concentration of 9.5% (w/v) was commonly used to provide the required condition of low conductivity (e.g., 6.9 μS cm^−1^) for ODEP operation. As discussed earlier, however, such a low conductivity environment might be lethal to biological cells because most biological activities (e.g., biochemical reactions) occurring within a cell are normally under high conductivity conditions [e.g., conductivity of cell cytoplasm: 2,000–5,000 μS cm^−1^ (Fatima H Labeed et al., 2003; Afshar et al., 2019)]. Nevertheless, the influences of the above sucrose solution on the viability of the manipulated cells and the performance of ODEP-based cell manipulation (e.g., the ODEP manipulation velocity or the percentage of the cells manipulatable by ODEP force) have generally been ignored in previous studies. To address this issue, experimental work was carried out. In this study, the viability of PC-3 cancer cells in normal (e.g., RPMI) cell culture medium (PRMI, DMEM, and F12K cell culture media are commonly used for general cell culture practice. They are similar in composition as shown in Supplementary Table S1) and in sucrose solution (9.5%) for a 4-h incubation time (e.g., 0, 2, and 4 h) was evaluated by using fluorescent dye staining and flow cytometry. The results (Figure 3A) showed that the viability of cancer cells in normal RPMI culture medium was maintained at a high level (i.e., 97.6 ± 0.2%, 97.8 ± 0.5%, and 96.2 ± 0.6% after 0, 2, and 4 h of incubation, respectively) and showed no significant difference (*p* > 0.05) after 4 h of incubation. Compared with these cell viabilities measured under normal culture medium conditions, however, the viability of cells in sucrose solution under the corresponding incubation time (e.g., 0, 2, and 4 h) conditions was measured to be significantly lower (*p* < 0.05). Moreover, the viability of cells in sucrose solution significantly (*p* < 0.05) declined during 4 h (95.3 ± 0.7%, 86.3 ± 0.7%, and 56.8 ± 1.5% after 0, 2, and 4 h of incubation, respectively), within which ODEP-based cell manipulation was generally performed. This phenomenon could be due to the aforementioned noncell-friendly environment of low conductivity (e.g., 6.9 μS cm^−1^), which could limit the biological activity within a cell (Yao and Asayama, 2017). This result could also be due to the sucrose solution-induced cell autophagy (Higuchi et al., 2015), which leads to cell death (Shimizu et al., 2014). Overall, the decrease in cell viability during the operation period of ODEP-based cell manipulation could greatly restrict the utilization of ODEP-based cell manipulation for biological research [e.g., cell isolation and purification for subsequent applications (Chiu et al., 2016; Liao et al., 2018; Chu et al., 2019a; Chu et al., 2020b)]. In this work, moreover, the strong cancer cell line (i.e., PC-3 cancer cells) was only tested. This abovementioned phenomenon will become a more important technical issue when some fragile cells (e.g., primary cells) are applied. This fact particularly highlights the importance of finding an appropriate background solution for ODEP-based cell manipulation.

In addition, the impact of sucrose solution on the performance of ODEP-based cell manipulation was also assessed experimentally. In this work, PC-3 cancer cells were cultured in normal RPMI cell culture medium and sucrose solution for 0, 2, and 4 h. Then, these treated cells were evaluated in terms of their ODEP manipulation velocity as well as the percentage of the cells manipulatable by ODEP force under a sucrose solution background. The results (Figure 3B) revealed that the maximum velocity of the light image that can manipulate the culture medium-treated cells (i.e., ODEP manipulation velocity) showed no significant difference (*p* > 0.05) during the 4-h incubation time (85.3 ± 18.9, 78.7 ± 26.7, and 76.7 ± 21.7 μm s^−1^ for 0-, 2-, and 4-h incubation, respectively). This trend was similar to the trend exhibited in the cell viability study of culture medium-treated cells (Figure 3A). In terms of the comparison between culture medium- and sucrose solution-treated cells, the ODEP manipulation velocity exhibited no significant difference (*p* > 0.05) except for the case of 4-h incubation time, in which the ODEP manipulation velocity of the sucrose solution-treated cells was significantly lower (*p* < 0.05) than the ODEP manipulation velocity of the culture medium-treated cells by 39.1%. Regarding the sucrose solution-treated cells, moreover, the ODEP manipulation velocity of the cells treated with such a solution within 2 h revealed no significant difference (*p* > 0.05) (88.4 ± 23.5 and 76.3 ± 24.1 μm s^−1^ for 0 and 2 h, respectively). After a 4-h incubation time, however, the ODEP manipulation velocity of the cells treated with sucrose solution decreased significantly (*p* < 0.05) by 38.8% in comparison with the ODEP manipulation velocity of the 2-h case (Figure 3B). Furthermore, a similar trend as the findings in Figure 3B was also observed in the measurement of the percentage of the cells manipulatable by the ODEP force, as shown in Figure 3C. In this evaluation, the percentage of the cells manipulatable by ODEP force for the 4-h sucrose solution-treated cells (i.e., 49.7%) significantly reduced (*p* < 0.05) by 33.9% compared with that of the 2-h sucrose solution-treated cells.

As a whole, the results in Figure 3 indicated that the ODEP-based cell manipulation using 9.5% (w/v) sucrose solution (Liao et al., 2018; Chu et al., 2019a; Chu et al., 2020a; Chu et al., 2020b) could significantly compromise the viability of the manipulated cells by 9.1 and 38.5% after 2 and 4 h of operation time in comparison with that at the initial stage of ODEP operation, respectively. Apart from the cell viability issue, prolonged operation time (e.g., 4 h) could significantly lead to inferior performance of ODEP-based cell manipulation including 47.2% reduction of ODEP manipulation velocity and 44.4% loss of the cells manipulatable by ODEP force in comparison with that at the initial stage of ODEP operation. This phenomenon could result from the increase in cell death under incubation in sucrose solution for 4 h, as observed in Figure 3A. In this situation, the cell membrane of PC-3 cancer cells was damaged, which might therefore have led to the loss of ions and thus a decrease in conductivity within the cells (i.e., $\varepsilon_{cell}^{*}$ of Eq. 2). This fact could accordingly attenuate the ODEP manipulation force acting on a cell (i.e., Re[*f*_*CM*_] of Eq. 1) and in turn the ODEP manipulation velocity based on Eqs 1–3, respectively. This phenomenon was also discussed in our previous study (Chu et al., 2019a; Chu et al., 2020a). Leveraging this technical feature, the phenomenon was also successfully applied for the sorting and isolation of cells with varied degrees of cell viability (e.g., for the purpose of drug resistance evaluation) (Chu et al., 2019a; Chu et al., 2020a). Moreover, membrane damage or deformation of a cell was also reported to be able to increase its interaction with the substrate surface on which it is ODEP manipulated (Chu et al., 2019b). Again, this phenomenon could adversely affect ODEP-based cell manipulation. In addition to the use of PC-3 cancer cells as the tested model, another cancer cell line (i.e., OECM-1 cancer cells) was also tested. The findings similar as the results in Figure 3 were exhibited in Supplementary Figure S2 (the descriptions of the results were provided in the Supplementary Material).

### Modification of Sucrose Solution for ODEP-Based Cell Manipulation

**FIGURE 4:**
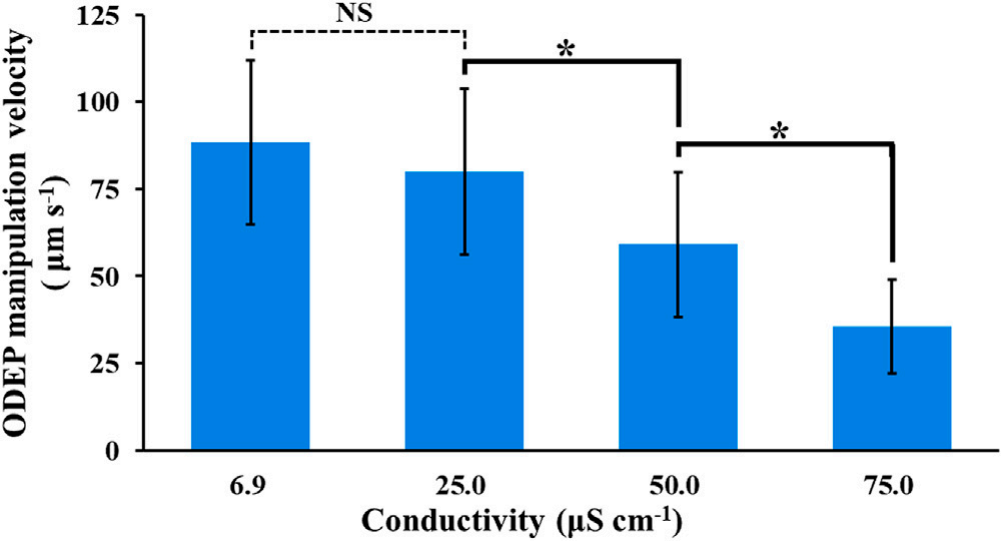
Evaluation of the ODEP manipulation velocity of PC-3 cancer cells under background solutions with varied conductivity (6.9–75.0 μS cm^−1^). The results are presented as the mean ± standard deviation of at least three separate experiments. [NS, no significance and *, significant difference (*p* < 0.05)].

The results in Figure 3 highlighted the importance of finding an improved background solution capable of improving the cell viability of the manipulated cells while maintaining the manipulation performance at a certain level during 4-h ODEP-based cell manipulation. To address this issue, the following investigations were carried out. First, the conductivity of the background solution is known to play a role in the ODEP force exerted on a cell (i.e., $\varepsilon_{m}^{*}\;$ of Eq. 2) (Valley et al., 2008) and thus its ODEP manipulation velocity (Eq. 3). To fundamentally understand to what extent the ODEP manipulation velocity is influenced by the conductivity of the background solution, the ODEP manipulation velocity of cells under sucrose solutions supplemented with various levels of PBS for modifying their conductivity (e.g., 6.9–100.0 μS cm^−1^) was experimentally evaluated. The evaluation was based on the processes described in Figure 2. The results (Figure 4) showed that the ODEP manipulation velocities (i.e., 88.4 ± 23.5 and 80.0 ± 23.9 μm s^−1^) of the cells in the PBS-supplemented sucrose solutions [i.e., measured conductivity: 6.9 and 25.0 μS cm^−1^, respectively] exhibited no significant difference (*p* > 0.05). When the conductivity of PBS-supplemented sucrose solutions was higher than 50 μS cm^−1^ (i.e., 50.0 and 75.0 μS cm^−1^), the measured ODEP manipulation velocity (i.e., 59.1 ± 20.9 and 35.6 ± 13.3 μm s^−1^, respectively) significantly (*p* < 0.05) declined. Moreover, in this evaluation, the cancer cells were hardly manipulatable when the conductivity of the background solution reached 100.0 μS cm^−1^ (result not shown). In this study, the ODEP manipulation velocity of cells under the conductivity condition of 50 μS cm^−1^ was 66.8% of the ODEP manipulation velocity in 9.5% sucrose solution (conductivity: 6.9 μS cm^−1^), which is acceptable for general ODEP-based cell manipulation. Based on the findings in Figure 4, therefore, the conductivity condition of lower than 50 μS cm^−1^ was then determined as a criterion for the following studies.

**FIGURE 5:**
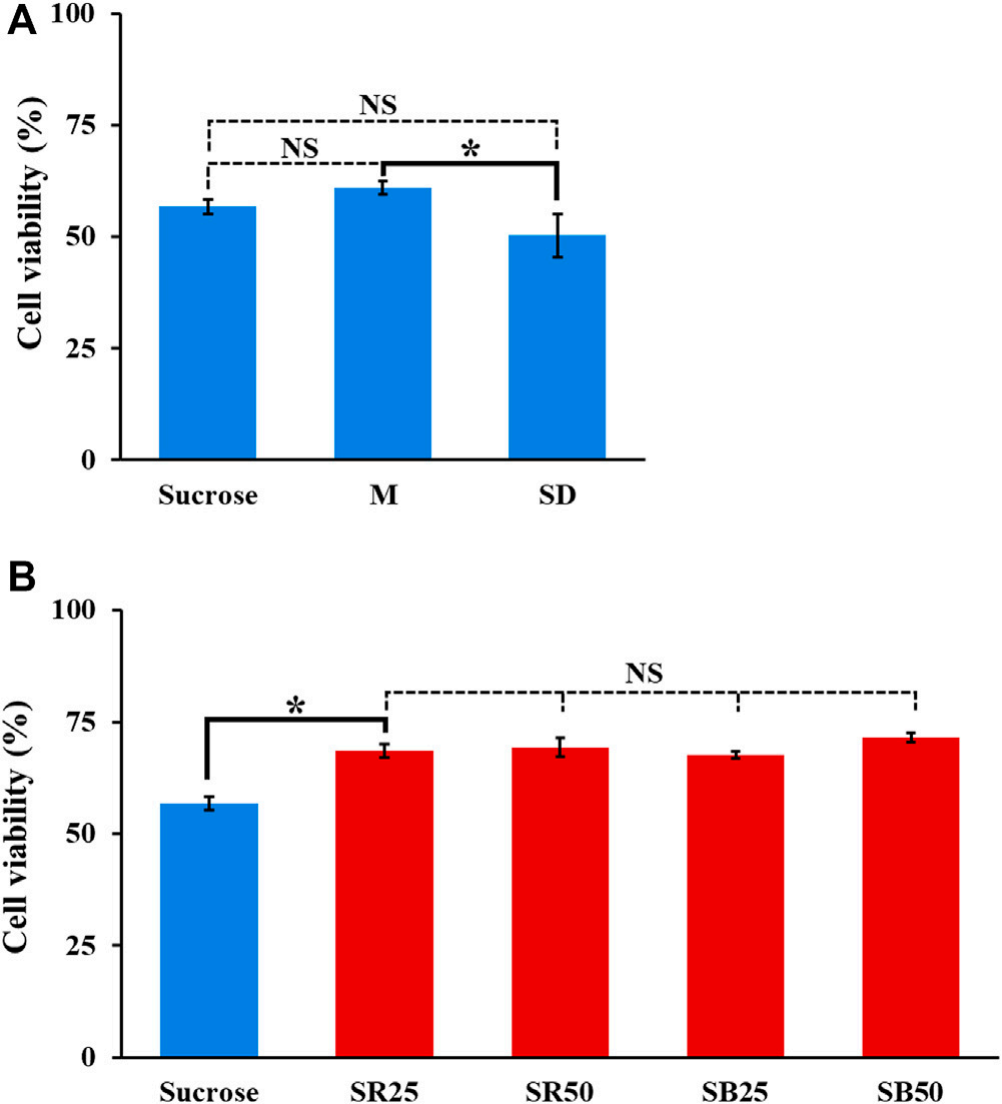
The cell viability (%) of PC-3 cancer cells incubated in the prepared background solutions with **(A)** lower and **(B)** higher conductivity for 4 h (M, SD, SR25, SR50, SB25, and SB50 are the background solutions prepared according to Table 1). The results are presented as the mean ± standard deviation of at least 3 separate experiments. [NS, no significance and *, significant difference (*p* < 0.05)].

In subsequent work, the modification of sucrose solution was carried out by supplementing ingredients to sucrose solution apart from the use of mannitol solution [5% (w/v)] as the background solution for the tests. In addition to the conductivity condition (i.e., lower than 50 μS cm^−1^) determined experimentally (Figure 4), the pH and osmolarity of the prepared background solutions with varied compositions were set at pH 6–7 and 270–300 mOsmol kg^−1^, respectively. These two condition ranges are commonly used in cell culture medium to prevent cell crenation, lysis, and death (Waymouth, 1970; von Euler et al., 2002). In this study, the supplemented ingredients included dextrose [0.3% (w/v)], PRMI cell culture medium [0.1 and 0.3% (v/v)], and BSA [0.25 and 0.5% (w/v)]. A low-conductivity mannitol solution was reported to be used as the background solution for DEP-based cell manipulation (Archer et al., 1999; Hoettges et al., 2007; L D'Amico et al., 2017), which might prevent the cell damage caused by the low conductivity sucrose solution commonly used for ODEP-based cell manipulation. Similarly, dextrose [e.g., 0.3% (w/v)] and BSA are commonly used as supplements in the background solution for DEP- or ODEP-based cell manipulation (Fatima H Labeed et al., 2003; Srinivasu Valagerahally Puttaswamy et al., 2010; Gupta et al., 2012; Song et al., 2015; Chiu et al., 2016). The former can serve as the energy source for biological cells (Yao and Asayama, 2017), and the latter is reported to prevent cell adsorption on the substrate surface, facilitating subsequent ODEP-based cell manipulation (Gupta et al., 2012; Song et al., 2015; Chiu et al., 2016), to reduce ROS (reactive oxygen species) production (Liu et al., 2012) and to prevent cell death (Liu et al., 2012). In addition, RPMI cell culture medium is commonly used for cell culture practice and can provide various basic nutrients (e.g., glucose or amino acids) for manipulated cells. Among these ingredients tested, supplementation with RPMI cell culture medium and BSA could easily cause an increase in solution conductivity. Therefore, supplementation in sucrose solution to form background solutions with a conductivity of approximately 25 and 50 μS cm^−1^ was arranged. Table 1 lists the background solutions prepared for the following tests to examine whether the use of these solutions could improve the viability of the manipulated cells while maintaining their manipulation performance at a certain level during 4-h ODEP-based cell manipulation. Figure 5A showed that the viabilities (50.3–61.1%) of the cells in the prepared background solutions with similar conductivity, osmolality, and pH values (i.e., mannitol solution, dextrose-supplemented sucrose solution, and sucrose solution) (Table 1) showed no significant difference in comparison with the cell viability of the original sucrose solution (*p* > 0.05) after 4 h of incubation. Within the experimental conditions explored, the results indicated that the use of mannitol or supplementation with dextrose could not reduce cell death under such a low conductivity culture environment. Conversely, the use of background solutions with higher conductivity (i.e., SR25, SR50, SB25, and SB50; Table 1) could significantly (*p* < 0.05) improve the cell viability by 10.9–14.8% in comparison with that of the original sucrose solution (cell viability: 56.8 ± 1.5%, 68.6 ± 1.6%, 69.4 ± 2.2%, 67.7 ± 0.8%, and 71.6 ± 1.0% for sucrose, SR25, SR50, SB25, and SB50 solutions, respectively) after 4 h of incubation (Figure 5B). This improvement in cell viability could be due to the increase in solution conductivity (i.e., from 6.9 to approximately 25 or 50 μS cm^−1^; Table 1), which could, to some extent, promote various biological activities (e.g., biochemical reactions) within a cell. Moreover, the improvement could also be due to the supplementation of basic nutrients for cell survival (i.e., the supplementation with RPMI culture medium) or BSA, which was reported to prevent cell death (Liu et al., 2012). Within the experimental conditions explored, the viability of cells in these modified sucrose solutions showed no significant difference (*p* > 0.05). Based on the findings in Figure 5, 9.5% (w/v) sucrose solutions supplemented with 0.25 and 0.5% (w/v) BSA (i.e., SB25 and SB50; Table 1) were selected for the following performance evaluation of ODEP-based cell manipulation.

### Performance Evaluation of ODEP-Based Cell Manipulation Using Modified Sucrose Solutions

**FIGURE 6:**
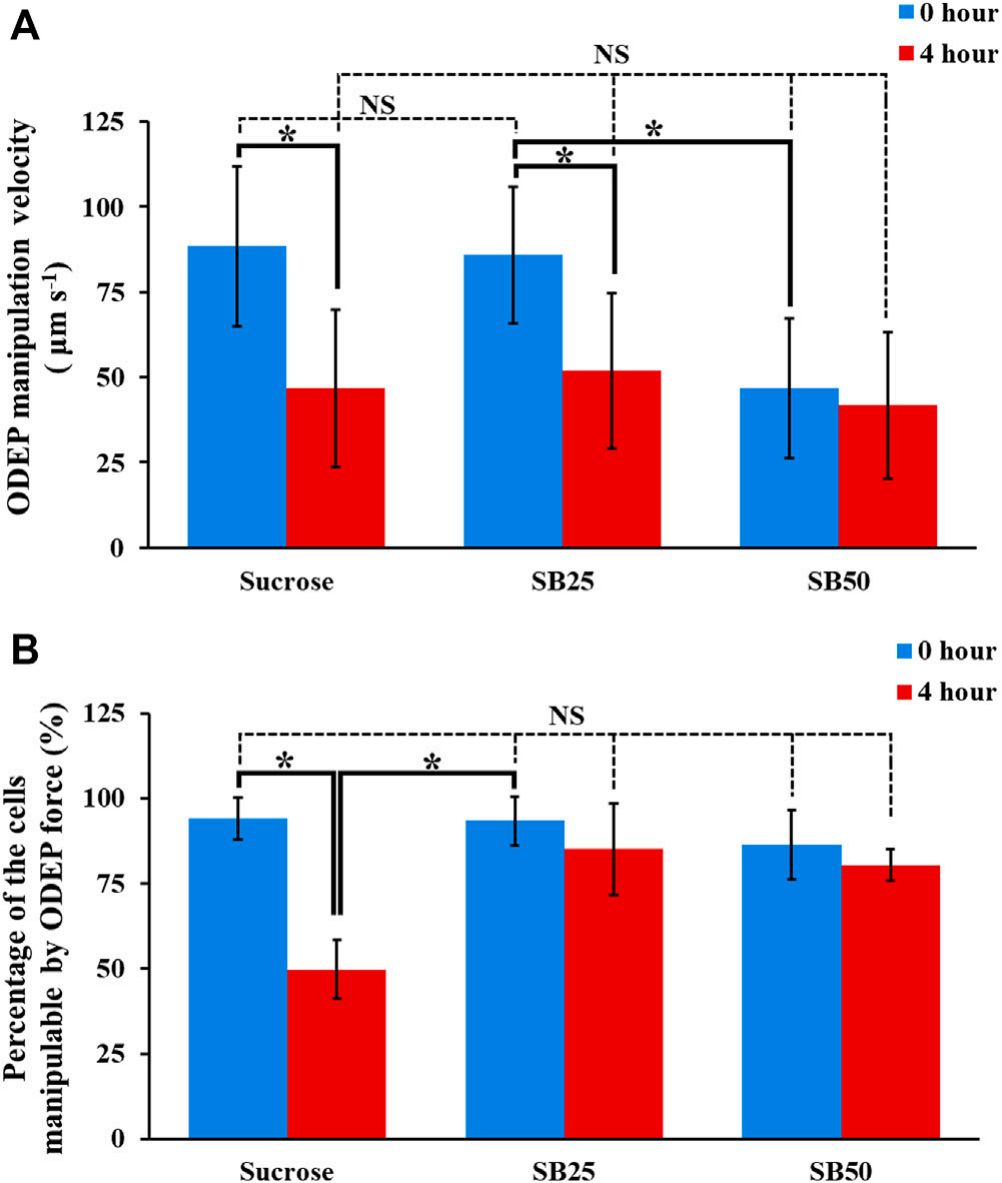
The **(A)** ODEP manipulation velocity and **(B)** percentage of the cells manipulatable by the ODEP force of the PC-3 cancer cells incubated in sucrose solution (9.5%), and sucrose solutions supplemented with BSA (SB25 and SB50; Table 1) for 0 and 4 h, respectively. The results are presented as the mean ± standard deviation of at least 3 separate experiments. [NS, no significance and *, significant difference (*p* < 0.05)].

Based on the study in Figure 5, the sucrose solution supplemented with BSA at different levels (i.e., SB25 and SB50; Table 1) was selected for the following evaluations in terms of the performances of ODEP-based cell manipulation within these two background solutions after 4-h incubation time. The results (Figure 6A) revealed that the initial (0 h) ODEP manipulation velocity of cancer cells in conventional sucrose (9.5%) and SB25 solutions showed no significant difference (*p* > 0.05) (measured ODEP manipulation velocity: 88.4 ± 23.5 and 85.9 ± 20.0 μm s^−1^ for sucrose and SB25 solutions, respectively), but the ODEP manipulation velocity significantly (*p* < 0.05) declined when the SB50 background solution was used (measured ODEP manipulation velocity: 46.7 ± 20.4 μm s^−1^). Overall, the ODEP manipulation velocity of cells in SB50 solution was significantly lower than the ODEP manipulation velocity of cells in SB25 solution by 45.7%. This finding was in line with the results in Figure 4, showing that the ODEP manipulation velocity of cells in the background solution with conductivity higher than 50 μS cm^−1^ significantly dropped compared with the lower conductivity counterpart. Except for the SB50 case, moreover, the ODEP manipulation velocity of cells at the beginning and after 4 h of incubation showed significant difference (*p* < 0.05). In the SB25 case, there was a 39.6% decrease in ODEP manipulation velocity after 4 h of incubation. In terms of the comparison of the ODEP manipulation velocity after 4 h of incubation, there was no significant difference (*p* > 0.05) among the cases explored (46.7 ± 23.1, 51.9 ± 22.6, and 41.8 ± 21.4 μm s^−1^ for sucrose, SB25, and SB50 solutions, respectively).

Apart from the ODEP manipulation velocity, more importantly, another critical performance factor of ODEP-based cell manipulation, which could determine the success of ODEP-based cell manipulation, is the percentage of the cells manipulatable by the ODEP force. The results (Figure 6B) showed that there was no significant difference (*p* > 0.05) in this factor among the cases studied at the initial stage (0 h) of ODEP-based cell manipulation (i.e., 94.2 ± 6.3, 93.5 ± 7.2, and 86.4 ± 10.2% for the sucrose, SB25, and SB50 solutions, respectively). Different from the 44.4% significant decrease in the sucrose solution (*p* < 0.05), this factor remained at a high level of 80.4–85.2% for the SB25 and SB50 solutions after 4 h of incubation, which showed no significant difference (*p* > 0.05) compared with their initial levels. This phenomenon could be explained by supplementation with BSA in sucrose solution being capable of significantly increasing the cell viability, as shown in Figure 5B. The increase in viable cells could accordingly increase the ratio of the cells manipulatable by the ODEP force (Chu et al., 2020a). In addition, BSA is generally believed to be able to prevent the adhesion of proteins (Liu et al., 2018) and thus cells on a surface (Liu et al., 2018), which could correspondingly facilitate ODEP-based cell manipulation. Taken together, overall, the results in Figure 6 suggest that the ODEP manipulation velocity of cells in the SB25 background solution is comparable to the ODEP manipulation velocity of cells in the conventional sucrose solution. The use of SB50 solution for ODEP-based cell manipulation could compromise the ODEP manipulation velocity by 45.7% in comparison with that of SB25 solution in the initial operation period. Moreover, compared with the conventional sucrose solution, the use of SB25 or SB50 solution could contribute to the high percentage (80.4–93.5%) of cells manipulatable by ODEP force during 4 h of operation. This phenomenon is critical for general ODEP-based cell manipulation. In addition to the use of PC-3 cancer cells as the tested model, another cancer cell line (i.e., OECM-1 cancer cells) was also tested. The findings similar as the results in Figures 4–6 were exhibited in Supplementary Figure S3 (the descriptions of the results were provided in the Supplementary Material).

## Conclusion

The ODEP technique is effective and user-friendly for fine cell manipulation. Nevertheless, the widespread utilization of this technique has been limited by the requirement of a background solution with low conductivity which is generally incompatible with the biological activity within a cell. This technical issue has been generally ignored in ODEP-relevant studies. To address this issue, this study aimed to investigate to what extent the cell viability of the manipulated cells and the performances of ODEP-based cell manipulation are affected by the background solution with low conductivity (e.g., 6.9 μS cm^−1^) in ODEP operation. Additionally, this study aimed to explore the possibility of modifying the sucrose solution to reduce the adverse impacts caused by the low conductivity of the background solution. The results revealed that the use of sucrose solution (9.5%) in ODEP operation could significantly reduce the viability of the manipulated cells by 9.1 and 38.5% after 2 and 4 h of incubation, respectively. Prolonged operation time (e.g., 4 h) in sucrose solution could significantly lead to inferior performances of ODEP-based cell manipulation including 47.2% reduction of ODEP manipulation velocity and 44.4% loss of the cells manipulatable by ODEP force compared with them in the initial stage of ODEP-based cell manipulation. The key finding of this study is that the use of BSA-supplemented sucrose solution with a conductivity range of 25–50 μS cm^−1^ might significantly increase the cell viability by 10.9–14.8% compared with the cell viability in sucrose solution after 4 h of incubation. Moreover, the ODEP manipulation velocity of cells in the BSA-supplemented sucrose solution (conductivity: 25 μS cm^−1^) was comparable to the ODEP manipulation velocity of cells in the conventional sucrose solution during a 4-h incubation. Compared with the use of conventional sucrose solution, more importantly, the use of BSA-supplemented sucrose solution (conductivity: 25–50 μS cm^−1^) could contribute to the high percentage (80.4–93.5%) of the cells manipulatable by ODEP force during 4-h ODEP operation, which showed no significant difference compared with that in the initial stage of ODEP-based cell manipulation in the sucrose solution. This performance is important and determines the success of ODEP-based cell manipulation. As a whole, this study has provided some fundamental information relevant to the improvement of background solutions for ODEP-based cell manipulation. Further study is required to explore the background solution with more complicated supplements that might be a step forward to improve both the cell viability and performance of ODEP-based cell manipulation.

## Data Availability

The original contributions presented in the study are included in the article/Supplementary Material, further inquiries can be directed to the corresponding author.
