# Supplementary material for: Improvement of Background Solution for Optically Induced Dielectrophoresis-Based Cell Manipulation in a Microfluidic System
Source: Front Bioeng Biotechnol. 2021 Nov 22;9:759205. doi: 10.3389/fbioe.2021.759205 (PMC8645848; doi:10.3389/fbioe.2021.759205)
Supplement: Supplementary file 1 [file DataSheet1.ZIP › Supplementary_Material P.Y. Chu.pdf]

Supplementary Material

1 The evaluation of cell viability and the performance of ODEP-based cell manipulation using OECM-1 cancer cell line as the test model (The description of the results in Fig. S2, S3, and S4)

In addition to the use of PC-3 cancer cells, OECM-1 cancer cell line (human oral squamous cancer cell line) was used as another test model in this study. The findings were similar as the results in the model of PC-3 cancer cells. Briefly, the results **[Fig. S2]** also showed that prolonged operation time (e.g., 4 h) in sucrose solution could lead to a significant decrease in the cell viability and performance of cell manipulation, including 27.7% reduction of cell viability, 58.3% loss of ODEP manipulation velocity, and 59.1% loss of the cells manipulatable by ODEP force. In addition, the results **[Fig. S3 (A)]** revealed that the ODEP manipulation velocities of OECM-1 cancer cells exhibited no significant difference ($p > 0.05$) in the solutions with the measured conductivity of 6.9 and 25.0 μS cm^{-1} but significantly ($p < 0.05$) declined when the measured conductivity was higher than 50.0 μS cm^{-1}. In subsequent work, therefore, the background solution with the measured conductivity lower than 25.0 μS cm^{-1} (i.e., M, SD, and SB25; Table 1) was selected for the following studies. After 4 h of incubation, the results **[Fig. S3 (B)]** indicated only SB25 solution could significantly ($p < 0.05$) improve the cell viability by 7.7% in comparison with that of the original sucrose solution. Based on the study in Fig. S3 (B), the sucrose solution supplemented with BSA (i.e., SB25; Table 1) was selected for the further performance evaluation of ODEP-based cell manipulation after 4-h incubation time. The results **[Fig. S3 (C)]** demonstrated the ODEP manipulation velocity of cells in the BSA-supplemented sucrose solution (conductivity: 25 μS cm^{-1}) was comparable with that in sucrose solution during 4-h incubation. Compared with sucrose solution, more importantly, the use of BSA-supplemented sucrose solution (conductivity: 25 μS cm^{-1}) could significantly improve the percentage of the cells manipulatable by ODEP force by 34.5% after 4-h incubation **[Fig. S3 (D)]**. In terms of microscopic observation, furthermore, it can be found that the cell membrane of sucrose solution-treated OECM-1 cancer cells become relatively irregular after 4 hr incubation. Conversely, the cell membrane of the cell culture medium or SB25 solution-treated OECM-1 cancer cells was still regular after 4 hr incubation **[Fig. S4]**. Taken together, the abovementioned results demonstrated that the use of BSA-supplemented sucrose solution (conductivity: 25 μS cm^{-1}) could significantly improve the cell viability and performance of ODEP-based cell manipulation.

2 Supplementary Figures

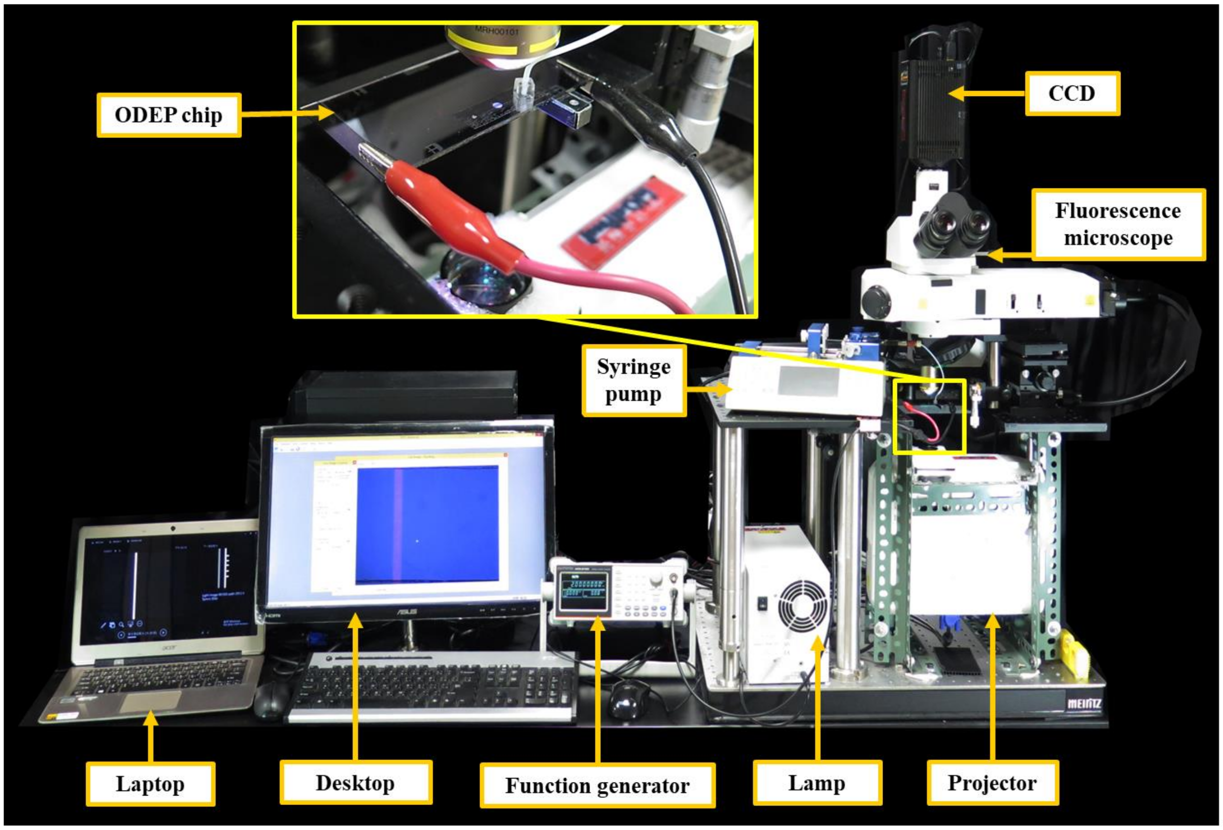

Figure S1. Photograph of the overall ODEP experimental setup.

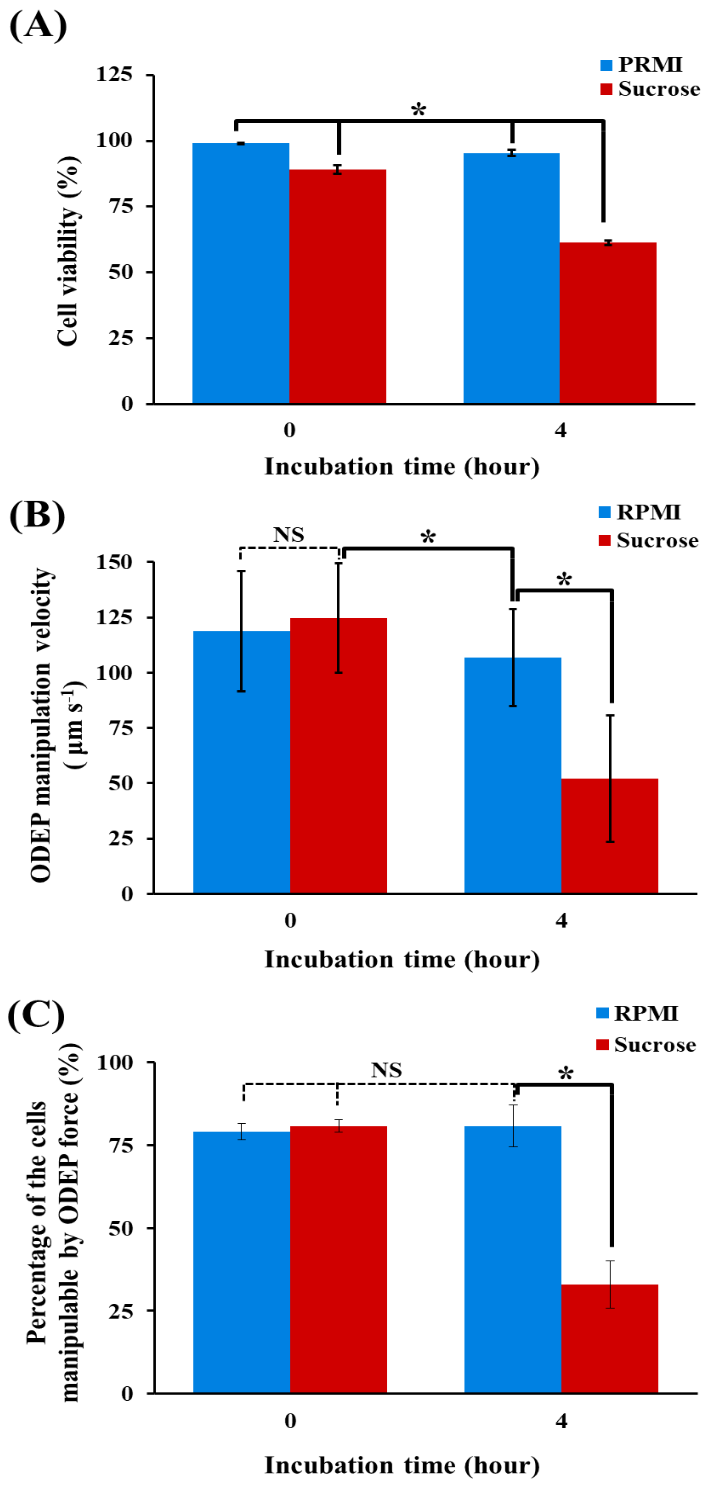

Figure S2. The **(A)** cell viability, **(B)** ODEP manipulation velocity, and **(C)** percentage of the cells manipulatable by ODEP force of the OECM-1 cancer cells treated with RPMI culture medium and sucrose solution (9.5%) for 0 and 4 h. The results are presented as the mean ± standard deviation of at least 3 separate experiments. (NS: no significance, and *: significant difference (p < 0.05)).

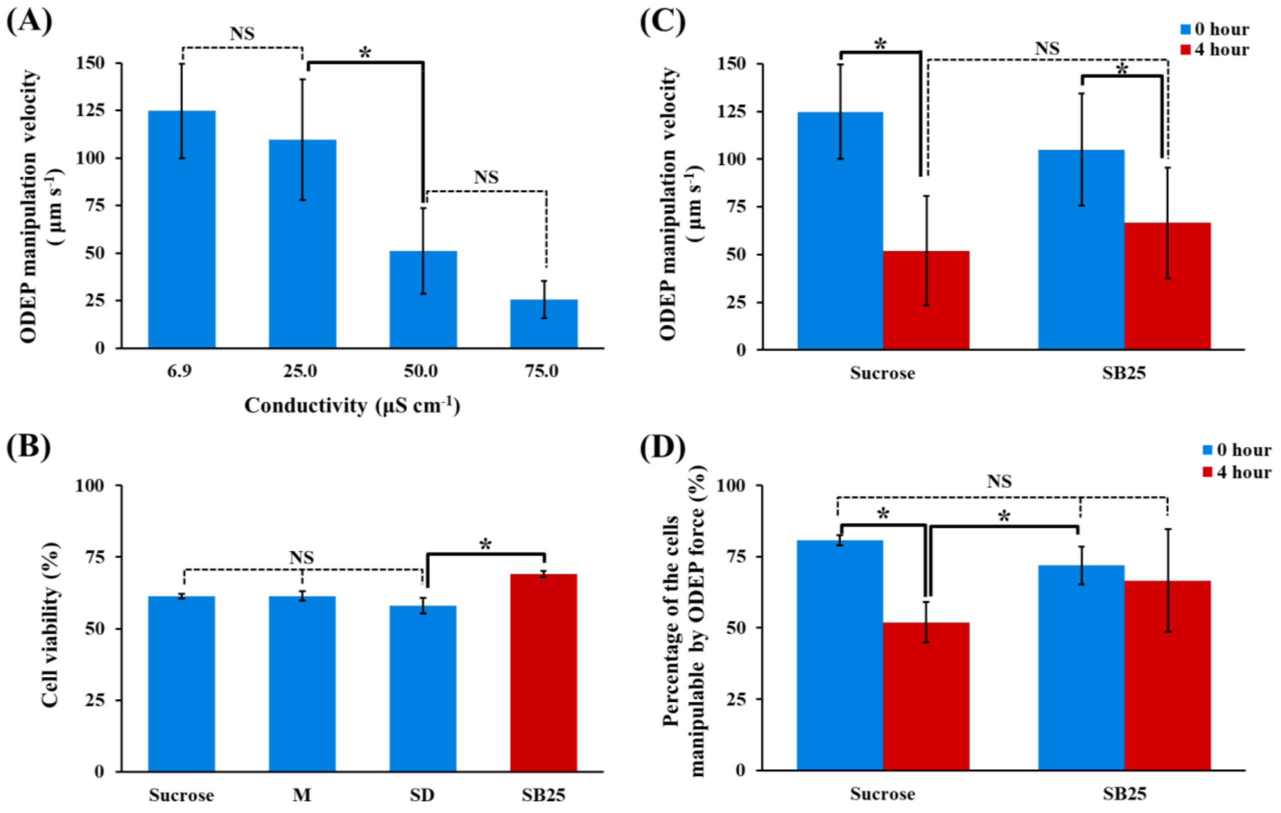

Figure S3. Evaluation of **(A)** the ODEP manipulation velocity of OECM-1 cancer cells under the background solutions with varied conductivity (6.9～75.0 μS cm^{-1}), **(B)** cell viability (%) of OECM-1 cancer cells incubated in the prepared background solutions (i.e., M, SD, and SB25 are the background solutions prepared according to Table 1) for 4h, as well as **(C)** the ODEP manipulation velocity and **(D)** the percentage of the OECM-1 cancer cells manipulatable by the ODEP force incubated in sucrose solution (9.5%) and sucrose solutions supplemented with BSA (SB25; Table 1) for 0 and 4 h, respectively. The results are presented as the mean ± standard deviation of at least 3 separate experiments. (NS: no significance, and *: significant difference ($p < 0.05$)).

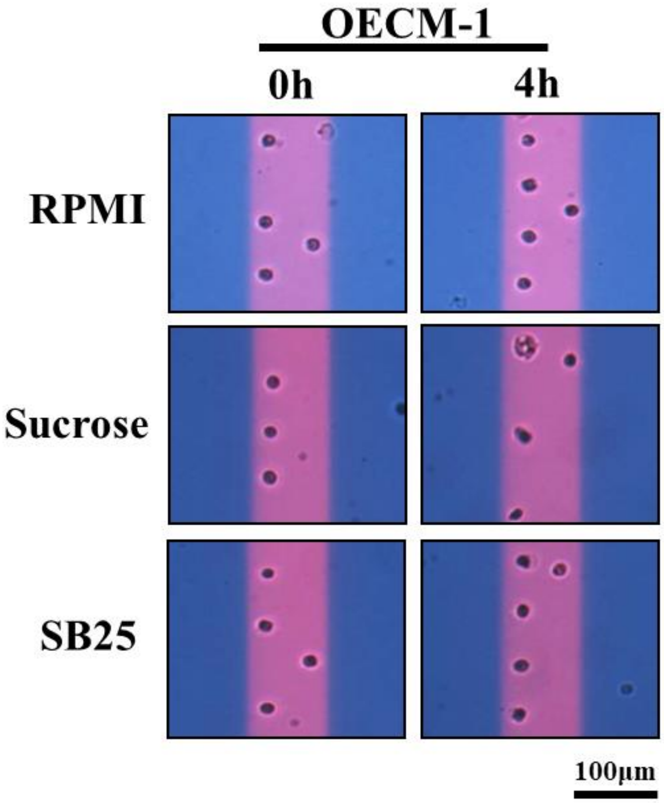

Figure S4. The photograph of OECM-1 cancer cells in the ODEP-based microfluidic chip for 0 or 4 hr incubation in RPMI, sucrose, or SB25 solutions (SB25 was the modified solutions prepared according to Table 1), respectively.

3 Supplementary Video

Video Clip: The operating procedures for the evaluation of "maximum velocity" of the dynamic light image that can manipulate cells (also called as "ODEP manipulation velocity")

4 Supplementary Table

Table S1. The composition comparison of RPMI, DMEM, and F12K culture media

Component	RPMI-1640*	DMEM*	F12K*
**Amino Acids**			
L-Cystine	✔	✔	✔
L-Glutamine	✔	✔	✔
Glycine	✔	✔	✔
L-Histidine	✔	✔	✔
L-Isoleucine	✔	✔	✔
L-Leucine	✔	✔	✔
L-Lysine	✔	✔	✔
L-Methionine	✔	✔	✔
L-Phenylalanine	✔	✔	✔
L-Serine	✔	✔	✔
L-Threonine	✔	✔	✔
L-Tryptophan	✔	✔	✔
L-Tyrosine	✔	✔	✔
L-Valine	✔	✔	✔
L-Arginine	✔	✔	✔
L-Alanine			✔
L-Asparagine	✔		✔
L-Aspartic Acid	✔		✔
L-Glutamic Acid	✔		✔
L-Proline	✔		✔
L-Hydroxyproline	✔		
**Vitamin**			
Choline chloride	✔	✔	✔
Folic Acid	✔	✔	✔
i-Inositol	✔	✔	✔
Niacinamide	✔	✔	✔
D-Pantothenic Acid	✔	✔	✔
Pyridoxine	✔	✔	✔
Riboflavin	✔	✔	✔
Thiamine	✔	✔	✔
Biotin	✔		✔
Vitamin B-12	✔		✔
Hypoxanthine			✔
p-Amino Benzoic Acid	✔		
Putrescine			✔
Thymidine			✔
**Inorganic Salt**			

$MgSO_4$	✔	✔	✔
KCl	✔	✔	✔
$NaHCO_3$	✔	✔	✔
NaCl	✔	✔	✔
Na_2HPO_4	✔	✔	✔
$Ca(NO_3)_2 \cdot 4H_2O$	✔		
$CaCl_2$		✔	✔
$Fe(NO_3)_3 \cdot 9H_2O$		✔	
$CuSO_4 \cdot 5H_2O$			✔
$FeSO_4 \cdot 7H_2O$			✔
$MgCl_2 \cdot 6H_2O$			✔
KH_2PO_4			✔
$ZnSO_4 \cdot 7H_2O$			✔
**Other**			
D-Glucose (Dextrose)	✔	✔	✔
Phenol Red	✔	✔	✔
Pyruvate			✔
Glutathione (reduced)	✔		
Lipoic Acid			✔

* The formulations of RPMI-1640 and DMEM culture media are obtained from ThermoFisher Scientific Inc. (https://www.thermofisher.com/tw/zt/home/technical-resources/media- formulation.114.html & https://www.thermofisher.com/tw/zt/home/technical-resources/media-formulation.8.html) and the formulation of F12K culture media is obtained from ATCC (American Type Culture Collection; https://www.summitpharma.co.jp/japanese/service/pdf/30-2004_F-12K.pdf).
